# Antibacterial, antibiofilm and cytotoxic activity of synthesized metal-incorporated mesoporous silica nanoparticles

**DOI:** 10.1186/s13568-025-01938-x

**Published:** 2025-09-17

**Authors:** Suzan Shehata, Yomna N. Elkholy, Mai S. A. Hussien, Ibrahim S. Yahia, Khaled M. Aboshanab

**Affiliations:** 1 Department of Microbiology and Immunology, Faculty of Pharmacy, Ain Shams University, Cairo, 11566 Egypt; 2 Department of Chemistry, Faculty of Education, Ain Shams University, Roxy, Cairo, 11757 Egypt; 3 Green Research Laboratory (GRL), Faculty of Education, Ain Shams University, Roxy, Cairo, 11757 Egypt; 4 Nanoscience Laboratory for Environmental and Bio-medical Applications (NLEBA), Faculty of Education, Ain Shams University, Roxy, Cairo, 11757 Egypt; 5 Laboratory of Nano-Smart Materials for Science and Technology (LNSMST), Department of Physics, Faculty of Science, King Khalid University, P.O. Box 9004, Abha, Saudi Arabia

**Keywords:** Multidrug-resistance, Nanoparticles, MRSA, Mesoporous silica nanoparticles, Antibiofilm activity, Cytotoxicity

## Abstract

Antibiotic misuse has triggered a global health crisis due to the rise of antimicrobial-resistant microorganisms. Recent studies suggest that nanoparticles (NPs), particularly silver nanoparticles, may provide a solution. However, their toxicity necessitates the development of novel carriers to enhance targeting while minimizing cytotoxicity. This study aimed to synthesize, characterize mesoporous silica nanoparticles (MSN) incorporated with silver alone or combined with zinc, and evaluate their antibacterial, antibiofilm, and cytotoxic properties. Characterization through X-ray diffraction (XRD) and scanning electron microscopy (SEM), revealed particle sizes between 413 and 560 nm. Antibacterial activity was assessed via the broth-dilution method against *Staphylococcus aureus* ATCC 25,923, *Escherichia coli* ATCC 25,922, Methicillin-resistant *Staphylococcus aureus* (MRSA) ATCC 33,591, and five multidrug-resistant pathogens (MDR). Two types of silver-incorporated mesoporous silica nanoparticles were synthesized: Ag/MCM-48 (10 mmol Ag) and Ag/Zn/MCM-48 (10 mmol Ag + 10 mmol Zn). The minimum inhibitory and minimum bactericidal concentrations ranged from 7.8 to 31.25 and 7.8 to 62.5 µg/mL, respectively. Ag/MCM-48 NPs exhibited 78% antibiofilm activity against MRSA ATCC 33,591, while Ag/Zn/MCM-48 NPs displayed up to 90% antibiofilm activity against MDR bacteria. Cytotoxicity assays revealed promising results, with CC_50_ values of 169.16 ± 6.43 µg/mL and 19.95 ± 0.63 µg/mL for Ag/Zn/MCM-48 NPs and Ag/MCM-48 NPs against *Vero* cell line, respectively. The IC_50_ values of Ag/Zn/MCM-48 and Ag/MCM-48 NPs were 62.17 ± 2.15 µg/mL and 3.58 ± 0.12 µg/mL against *Caco*-2 cell line, respectively. Accordingly, our synthesized silver/zinc incorporated mesoporous silica NPs present a safer antibacterial and antibiofilm agent with lower cytotoxicity than silver NPs, making them a promising alternative for combating MDR bacteria.

**Supplementary Information:**

The online version contains supplementary material available at 10.1186/s13568-025-01938-x.

## Introduction

Antimicrobial resistance (AMR) has been identified as one of the top 10 global public health crises by the World Health Organization (WHO, 2023). The United Nation General Assembly warned in 2016 that unchecked AMR progression could lead to catastrophic consequences by 2050 (Hochvaldová et al. 2022). AMR arises when microorganisms evolve unresponsiveness to previously effective antimicrobial agents (EClinicalMedicine 2021; Tang et al. 2023). The WHO’s 2024 report highlights a significant increase in high-threat pathogens (Kuznetsova, et al., 2025). Key drivers of AMR include: (1) Overuse and misuse of antibiotics in healthcare and agriculture (Wan Mohtar et al. 2023; Aloke et al. 2025). (2) Prolonged exposure to disinfectants. (3) Stagnation in antibiotic innovation, leading to massive consumption of old empirical antibiotics, which escalates the antibiotic resistance crisis (Chen et al. 2023). According to the Centers for Disease Control and Prevention (CDC), third-generation cephalosporins no longer affect Gram-negative bacteria. *E. coli* (mutant strain) turned out to be resistant to carbapenem (Saif et al. 2023). The biofilm-producing bacteria exacerbate resistance by sheltering within extracellular matrices, requiring higher antibiotic doses than their planktonic counterparts that risk toxicity (Ugalde-Arbizu et al. 2023). The ESKAPE pathogens are notorious for hospital-acquired infections and adaptive virulence (da Rosa et al. 2020). One of the most famous microorganisms of the ESKAPE group is *S. aureus*, which is considered a main part of the skin microbiome, but with the rise of antibiotic-resistant strains involving the well-known MRSA, it has become a main source of nosocomial infections (Church and Mckillip 2021).

In the direction of addressing ways to overcome AMR, it is important to understand the underlying resistance mechanisms. Resistance can occur through several means such as antibiotic inactivation by microbial enzymes produced, limited antibiotic penetration of antibiotics due to decreased cell permeability, activation of efflux pump that expels antibiotics and target bypass as the bacteria produces proteins that facilitate its resistance like the penicillin binding protein PBP-2 A, that causes methicillin resistance in *S. aureus* (Pulingam, et al., 2022); Zhu et al. 2022). The production of new antibiotics is time-consuming, so finding cheap, safe, and quick synthesis alternatives to treat microbial infections is a must, such as nanoparticles (Banoub et al. 2021; Morgan and Aboshanab 2024). Nanoparticles are suitable alternatives because of many factors, including their size, stability, morphology, high loading capacity (Basavegowda and Baek 2021), and targeted antibiotic delivery (Alfatemi et al., 2018). The main mode of action of nanoparticles against MDR bacteria is the production of reactive oxygen species (ROS) (Horie and Tabei 2021), penetrating the bacterial cell membrane and interacting with the intracellular proteins and DNA as well as the electrostatic interaction, causing its damage (Fahim et al. 2024; Raghunath and Perumal 2017; Abuzeyad et al. 2025; Staroń and Długosz 2021). Nanoparticles can be synthesized by three main approaches: the top-down approach (the physical method) and the bottom-up approach (the biological or chemical method) (Altammar 2023).

There are different types of nanoparticles, including carbon-based nanoparticles such as graphene oxide, which exhibit promising features for medical applications (El-Khawaga et al. 2022), (Abuzeyad et al. 2023), organic NPs (e.g., polymeric NPs), and inorganic nanoparticles (ceramic NPs, semiconductor NPs, metal NPs, and metal oxide nanoparticles) (Hamad et al. 2020). Silver nanoparticles (Ag NPs) demonstrate exceptional antimicrobial activity due to their unique physicochemical characteristics, such as low volatility, cost-effectiveness, eco-friendliness, and high thermal stability (Hamad et al. 2020). In addition to their antimicrobial effects, Ag NPs have anti-inflammatory and anti-angiogenesis properties (Bidaki et al. 2025). However, their practical applications are restricted by their oxidation-induced aggregation, reduced antimicrobial activity, and their considerably high toxicity. To address these challenges, AgNPs can be stabilized using carriers to provide more safety and prolong the antimicrobial activity, such as mesoporous silica nanoparticles (MSN) (Liu et al. 2019; Mohamed Isa et al. 2021; Montalvo-Quirós et al. 2021). Mesoporous silica nanoparticles (MSN) were first discovered by the Mobil Oil Research group. They are porous solid materials. MSNs were first used as nanocarriers in 2001 by Vallet-Regí et al. for ibuprofen delivery. MSNs have been classified as generally recognized as safe (GRAS) by the FDA for nearly 50 years (Mohamed Isa et al. 2021).

The porous structure of MSN has cavities that can accept and release a lot of biomolecules and other therapeutic agents (Ahmed et al. 2025). In this sense, mesoporous silica NPs can be synthesized with variable particle diameter and pore diameter, with other nanoparticles incorporated into their network (Manzano and Vallet-Regí 2020). In this study, silver-incorporated MSNs (Ag/MCM-48) and silver-zinc co-incorporated MSNs (Ag/Zn/MCM-48) were synthesized. Zinc was selected for its dual role as an essential trace element for vital cellular reactions at low concentrations (Pasquet et al. 2014) and its potent antioxidant and antibiofilm activity (Saif et al. 2021). Zinc exerts bactericidal effects via the Reactive Oxygen species (ROS) mechanism and increases cell permeability, causing cell distortion (Czyżowska and Barbasz 2022; El-Saadony et al. 2024; Kim et al. 2022). Therefore, silver and zinc incorporated mesoporous silica NPs and silver incorporated mesoporous silica NPs were synthesized to investigate their synergistic antimicrobial activity against both Gram-positive and Gram-negative bacteria. This study also aimed to compare their cytotoxic effect and antibiofilm activities, specifically examining how the incorporation of zinc influences antibacterial activity and its potential role in decreasing the cytotoxicity associated with silver.

## Materials and methods

### Chemicals and reagents

Cetyltrimethylammonium bromide (CTAB), citric acid, ammonia, ethanol 95%, tetraethyl orthosilicate (TEOS), silver nitrate (AgNO3), and zinc nitrate (ZnNO3) were all purchased from Alpha Chemika, India. Nutrient agar media was obtained from Lab M, England. Gentamicin was bought from Memphis Co. Cairo, Egypt, while ciprofloxacin was provided by Amriya Co., Egypt. Tryptic soy broth (TSB) was supplied by Difco Laboratories, Detroit, USA. Crystal violet was provided by EL-Nasr Pharmaceutical Chemicals Co. (ADWIC), Qalyubia, Egypt. Dimethyl sulfoxide (DMSO) 0.5% and MTT (3-(4,5-dimethylthiazol-2-yl)−2,5-diphenyltetrazolium bromide) were provided by Sigma (St. Louis, MO, USA). Fetal Bovine serum, RPMI-1640 (Roswell Park Memorial Institute, HEPES (4-(2-hydroxyethyl)−1-piperazineethanesulfonic acid) buffer solution, L-glutamine, DMEM (Dulbecco’s modified Eagle’s medium), and 0.25% Trypsin-EDTA were purchased from Lonza (Belgium). The *Caco*−2 cell line (intestinal carcinoma cell line) and the *Vero* cell line (Mammalian cells from African Green Monkey Kidney) were both provided by the American Type Culture Collection (ATCC, Rockville, MD, USA).

### Synthesis of mesoporous silica nanoparticles (NPs)

#### Synthesis by a homogeneous system with CTAB

Cetyltrimethylammonium bromide (CTAB) 2.5 g was added directly to 50 mL of deionized water. Using a magnetic stirrer, the powder dissolved to get a solution. Then aqueous ammonia (13.2 mL) and absolute ethanol (60 mL) were added, respectively. Whilst the mixture was continuously stirred by the magnetic stirrer, Tetraethyl orthosilicate (TEOS) (4.6 mL) was added dropwise over 15 min to form a gel. The measured pH level was 10. After 2 h of stirring at room temperature, the white precipitate was collected with filter papers, followed by washing several times with distilled water (Venkatathri 2007).

#### Synthesis by a heterogeneous system with CTAB

At room temperature, cetyltrimethylammonium bromide (CTAB) 2.5 g powder was set to be dissolved in 120 mL of distilled water by a magnetic stirrer. After that, aqueous ammonia (9.5 mL) was added to the produced solution, followed by the dropwise addition of 10 mL of TEOS while continuously stirring for 15 min, adjusting the pH level to 10. The whole mixture was subjected to stirring for 1 h. After that, the produced white precipitate was filtered with filter papers and then washed three times with distilled water. The precipitate was dried in an oven at 90 °C, then calcined for 8 h at 550 ˚C (Venkatathri 2007).

### Synthesis of mobile composition of matter no. 48 (MCM-48) NP

A solution of cetyltrimethylammonium bromide, 2.4 g, and 50 mL of distilled water was formed with the assistance of a magnetic stirrer. Ethanol (50 mL) was added while stirring. Then, 12 mL of ammonia was added and stirred for 10 min. Dropwise, TEOS (3.4 g) was added at a pH level of 10. At room temperature, the total mixture was constantly stirred for 2 h. As in the two previously mentioned procedures, the produced precipitate was subjected to filtration and washing with distilled water thrice, followed by air drying for 24 h. At the end, the final powder was exposed to calcination at 550 °C for 6 h (Kumar et al. 2001).

### Synthesis of Ag/MCM-48 NPs and Ag/Zn/MCM-48 NPs

One-gram MCM-48 was added to a solution of 20 mmol of citric acid, 10 mmol AgNO_3_ (Alpha chemicals) alone (to produce Ag/MCM-48 10 mmol NPs) or with 10 mmol zinc nitrate (to produce Ag/Zn/MCM-48 10 mmol NPs) dissolved in deionized water at pH level 10, then stirred for 24 h by magnetic stirrer at room temperature. The yielded precipitate was collected by filtration, washed with both water and ethanol 95% three times, dried overnight at 120 °C, and calcined for 4 h at 400 °C (Tavakoli et al. 2019).

### Characterization of the synthesized nanoparticles

X-ray diffraction (XRD) was employed as a technique to investigate the synthesized nanoparticles’ structure using a X-beam diffractometer (XRD, D8-Find, Bruker, with *CuK*_*α*_ radiation (1.5418 A), Madison, WI, USA). Working at a current of 40 MA, a voltage of 40 kV, and a step filter of 0.01°. Origin Pro software was used to convert the produced data into peaks. The identification of the crystal structure of the synthesized NPs was done by X’pert High Score software. High-resolution images of the synthesized samples were obtained by scanning electron microscopy (SEM) using Quanta FEG 250 scanning electron microscope (FEI Company, Hillsboro, Oregon-USA). The synthesized nanoparticles were set on SEM stubs, with a 10.1 mm working distance, an in-lens detector with an excitation voltage of 20 kV. providing insights into the surface characteristics of NPs. Measurements of the grain size of the synthesized NPs were resolved by the Smile View software. Scherrer’s equation was used to calculate the crystalline size (Jahan Tamanna et al. 2024) as follows:

$$\:D=\frac{0.9\:\lambda\:}{\beta\:{cos}\,\theta}$$

The crystallite size is D, the X-ray wavelength is λ, β is the full width at half maximum (FWHM), and ϴ is the diffraction peak angle.

Dislocation density (δ) was attained via:

$$\:\delta\:=\frac{1}{{D}^{2}}$$

Lattice strain (ε) was calculated by:

$$\:\epsilon\:=\frac{\beta\:\text{cos}\,\theta}{4}$$

### Evaluating the antibacterial activity of the synthesized nanoparticles

#### Bacterial isolates

##### Reference strains

Reference bacterial strains were used for testing the antibacterial activity of the synthesized NPs. The tested strains were *S. aureus* ATCC 25,923 and E. *coli* ATCC 25,922. They were kindly provided by the Egypt Regional Center for Mycology and Biotechnology (RCMB), Al-Azhar University, Cairo, Egypt.

### Clinical pathogenic multidrug-resistant isolates (MDR)

Five clinical MDR bacterial isolates, including *K. pneumoniae* 36 AK and 78 TE, *E. coli* 48 WE and 25 LN, *S.* Typhi 24 ZA isolates and standard MRSA ATCC 33,591 were provided by the Ain Shams University culture collection (CCASU), Cairo, Egypt.

### Agar well diffusion

The antibacterial activity against reference strains was performed using the agar well diffusion method. Strains were cultured overnight at 37 ˚C (Balouiri et al. 2016). A single colony of each was used to prepare a fresh inoculum (0.5 McFarland) and surface inoculated over two nutrient agar plates (Lab M, England). Wells were punched using a sterile cork-borer (6 mm). One hundred microliters of each synthesized NPs (dissolved in deionized water with sonication to make a concentration of 10 mg/mL for each sample) were added to the wells and incubated overnight (in triplicate). Gentamicin 4 µg/mL (Memphis Co., Egypt) was used as a control, and sterilized distilled water as a blank. Then, the agar plates were incubated overnight at 37 ˚C (Al-Momani et al. 2024; Hossain et al. 2022). The antibacterial activity against MDR organisms was performed using agar well diffusion method as described previously, except that the cup diameter was 10 mm, and the positive control was either gentamicin concentration was 4 µg/mL or ciprofloxacin at 10 µg/mL (Amriya Co., Egypt) (Magaldi et al. 2004).

### Minimal inhibitory concentrations of the synthesized nanoparticles

The synthesized NPs were further tested against the MDR pathogens to find their minimum inhibitory concentrations (MICs). According to the Clinical & Laboratory Standards Institute CLSI, 2023, the MICs of the NPs were determined using the broth microdilution method (CLSI 2023). Each well of 96 round-bottom microtiter plates was first filled with 100 µL TSB. Stock solution: 10 mg of each sample was suspended in 10 mL sterile distilled water (1000 µg/mL). About 100 µL of the synthesized NPs were added to the first well in the row and serially diluted two-fold. Bacterial suspension of the test organism (0.5 McFarland) was prepared, and 10 µL of the prepared suspension was added to each well in three replicates, within 15 min, except for the negative control wells. The microtiter plate was incubated at 37 ˚C for 18–20 h in ambient conditions. Following incubation, MIC was determined (the lowest concentration of the NPs in the dilution series showing no visible growth of the test organism) (Magaldi et al. 2004).

### Minimum bactericidal concentration

To know the Minimum Bactericidal Concentration (MBC), the dilution that shows the Minimum Inhibitory Concentration (MIC) and two higher concentrations were plated, incubated at 37 ˚C for 24 h, and the viable cells were counted (CFU/mL) (CLSI 2023). The lowest concentration showing no bacterial colonies is defined as the MBC (van Hengel et al., 2020).

### In vitro antibiofilm activity

The antibiofilm activity of half MIC of the synthesized NPs against four strong biofilm producers, MRSA ATCC 33591, *E. coli* 25 LN, *K. pneumoniae* 78 TE, and *S.* Typhi 24 ZA was performed. A microtiter plate assay was conducted. Each well of a 96-well flat-bottom plate was inoculated with 100 µl of fresh trypticase soy yeast broth (TSB) (Difco Laboratories, Detroit, USA) containing 10^6^ CFU/mL of the test organism and sublethal concentrations of the synthesized NPs (50% of MIC) in triplicate. Control wells contained sterile media. The plates were incubated for 48 h at 37 ˚C. After incubation, the supernatant was discarded, and each well was rinsed with sterile distilled water to remove any free-floating cells. The plates were subsequently air-dried for 30 min. Aqueous solution of crystal violet 0.1% (EL-Nasr Pharmaceutical Chemicals Co. (ADWIC, Qalyubiah, Egypt) was added to each well and incubated for 15 min. The excess stain was removed; the wells were washed three times using sterile distilled water (van Hengel et al., 2020). Finally, the dye that adhered to the cells was solubilized by adding 250 µl of 95% v/v ethanol, followed by a 15-minute incubation. Absorbance was then measured spectrophotometrically at 590 nm with a microplate ELISA reader (Platos R496 Microplate reader, AMD diagnostics, Graz, Austria). To determine the percentage of biofilm change, antibiofilm activity was calculated as follows:

Percentage change in biofilm = A _590_ (untreated control) − A _590_ (treated with NPs)/A _590 _control ×100 (Wu, et al., 2020).

### Assessment of cytotoxic activity

The cytotoxic activity of the synthesized NPs was conducted using the MTT assay (3-(4,5-dimethylthiazol-2-yl)−2,5-diphenyltetrazolium bromide, Sigma-Aldrich). This assay assessed the cytotoxic effects of the synthesized NPs against the Vero cell line and the Caco-2 colon cancer cell line. For the Vero cell line, the cells were cultured in Dulbecco’s Modified Eagle Medium (DMEM), enriched with 10% heat-inactivated fetal bovine serum, 1% L-glutamine, HEPES buffer, and gentamicin (50 µg/mL). The Caco-2 cells were maintained in Roswell Park Memorial Institute (RPMI-1640) medium with 10% inactivated fetal calf serum and 50 µg/mL gentamicin. The cells were incubated at 37 ˚C with 5% CO_2_ in a humid environment and passaged two to three times weekly. Cells were diluted in the medium to a concentration of 1 × 10^4^ cells per well for the *Vero* cell line and 5 × 10^4^ cells per well for the *Caco*−2 cell line in 96-well sterile flat-bottom tissue culture plates (Corning^®^, London, UK), then incubated for 24 h. Afterward, the NPs were added in three replicates at serial two-fold dilutions. Control groups used were six wells with media or 0.5% DMSO for each 96-well plate. The plates were incubated at 37 ˚C in humid conditions with 5% CO_2_ for another 24 h and the numbers of viable cells were measured using the MTT test. Optical density was determined using a microplate reader (SunRise, TECAN, Inc., USA) (Singh et al. 2021).

### Statistical analysis

All the experiments were performed in triplicate, and results were expressed as mean values. The standard deviations were graphed as error bars using Microsoft Excel 2021 (USA). The overall methodology is displayed in Fig. S1.

## Results

### Characterization of the synthesized NPs

#### X-ray diffraction measurements and analysis of the synthesized nanoparticles

**Fig. 1:**
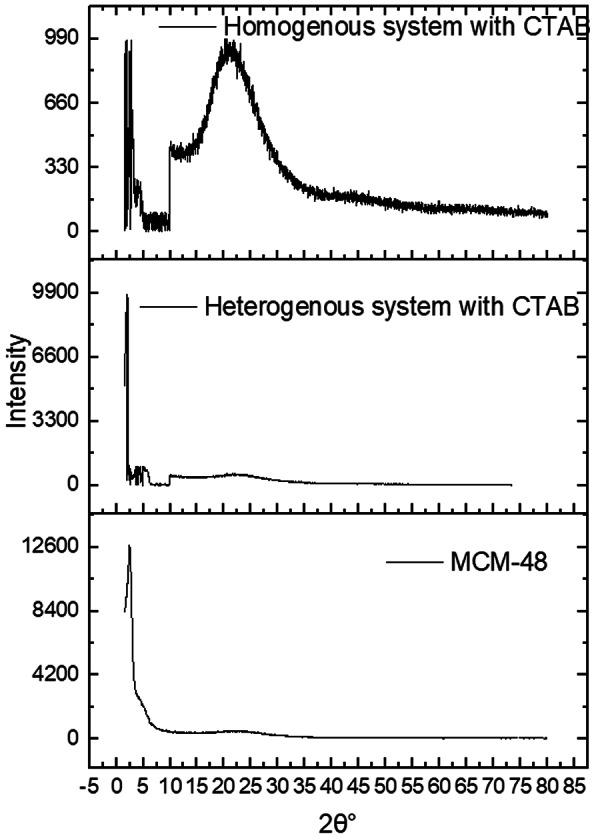
The X-ray diffraction (XRD) analysis of: MCM-48 NPs, MSNs by heterogeneous system and MSNs by homogenous system

**Fig. 2:**
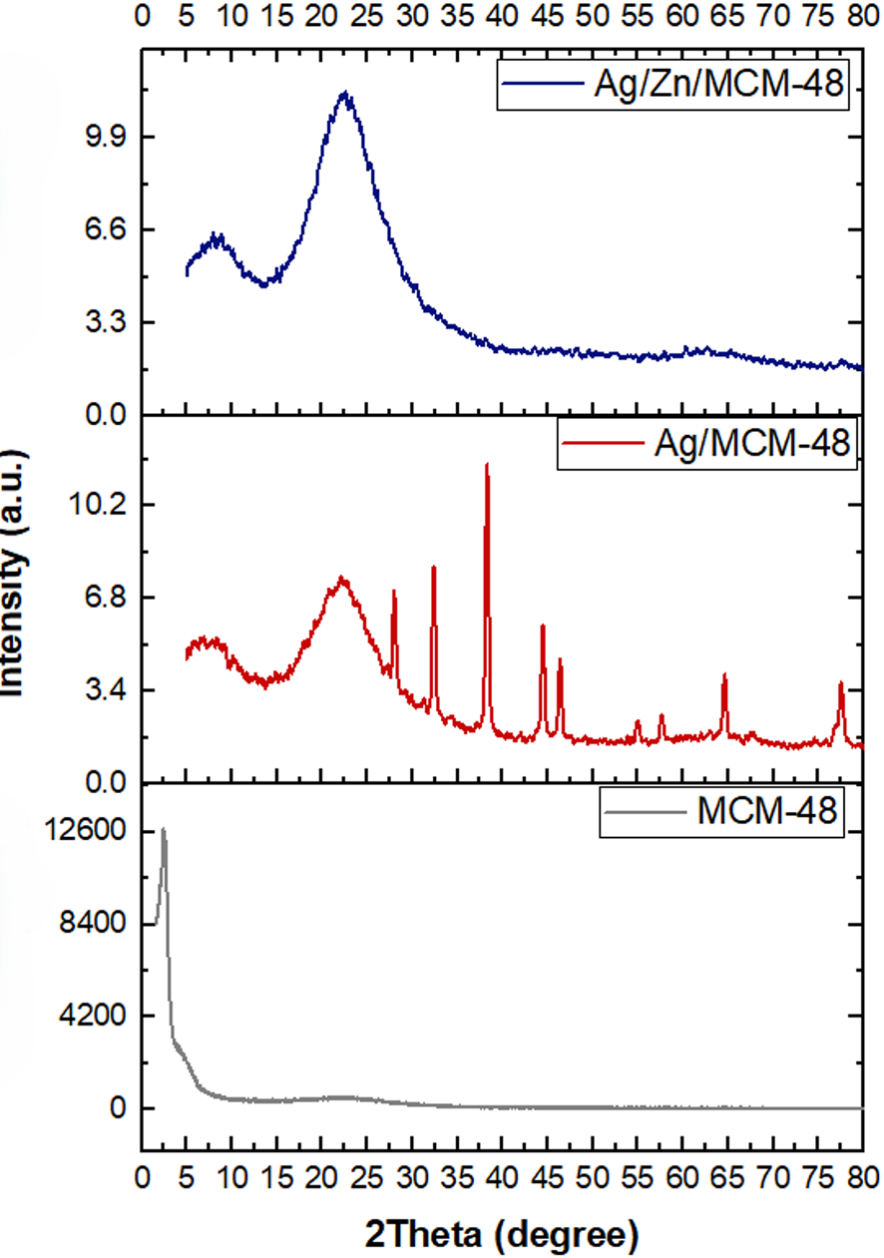
The X-ray diffraction (XRD) analysis of: MCM-48 NPs, Ag/MCM-48 NPs and Ag/Zn/MCM-48 NPs

The crystallinity assessment of the synthesized nanoparticles was performed by XRD. Using Scherrer’s equation to calculate the crystallite size. The results have been tabulated in Table 1. MSNs (homogenous system) showed a diffraction peak at 2θ equals to 21.64°, while MSNs (heterogeneous system) showed diffraction peaks at 2θ equals to 21.88° and 10.03°. Pure MCM-48 NPs showed a diffraction peak of plane (211) at 2θ equals to 2.37°, affirming the production of well-ordered Ia3d cubic structure in accordance with previous literature (Kalita et al. 2024), (Yismaw et al. 2023). The XRD results of the three MSNs are depicted in Fig. 1. Ag/MCM-48 10 mmol NPs showed multiple diffraction peaks of planes (111), (200), (220) and (311) at 2θ equal 38.24°, 44.57°, 64.6°, and 77.5°confirmed the silver’s crystalline phase and cubic structure which agrees with Tian et al., (Tian et al. 2021) representing the pattern of Joint Committee on Powder Diffraction Standards (JCPDS, No. 4-0783), which consistent with Hudaya et al., (Hudaya et al. 2024). The broad peak of Ag/Zn/MCM-48 NPs at 2θ equals 22.75° may be attributed to the amorphous nature of silica, and zinc was successfully loaded on MCM-48, and its size was too small to be detected by XRD (Shen et al. 2018). The weak intensity of the characteristic silver peaks may be attributed to the well-dispersion of silver in MCM-48 NPs (Es-haghi et al. 2024). The XRD results of MCM-48, Ag/MCM-48 (10 mmol), and Ag/Zn/MCM-48 (10 mmol) NPs are depicted in Fig. 2.

**Table 1:** Crystallite domain size, dislocation density and lattice strain results of the prepared NPs

Prepared NPs	Mean values crystallite domain size D, (nm)	Mean values of dislocation density (δ, (nm)	Mean values of lattice strain(ε)
MSNs (homogenous system)	205.3822	2.371E-05	1.688E-04
MSNs (heterogenous system)	152.687	5.9445E-05	2.541E-04
MCM-48	101.0168	9.800E-05	3.431E-04
Ag/MCM-48 10 mmol	102.66	3.79975E	3.28725
Ag/Zn/MCM-48 10 mmol	86.8239	5.7865E-04	4.133E-04

### Scanning electron microscope analysis of the synthesized nanoparticles

**Fig. 3:**
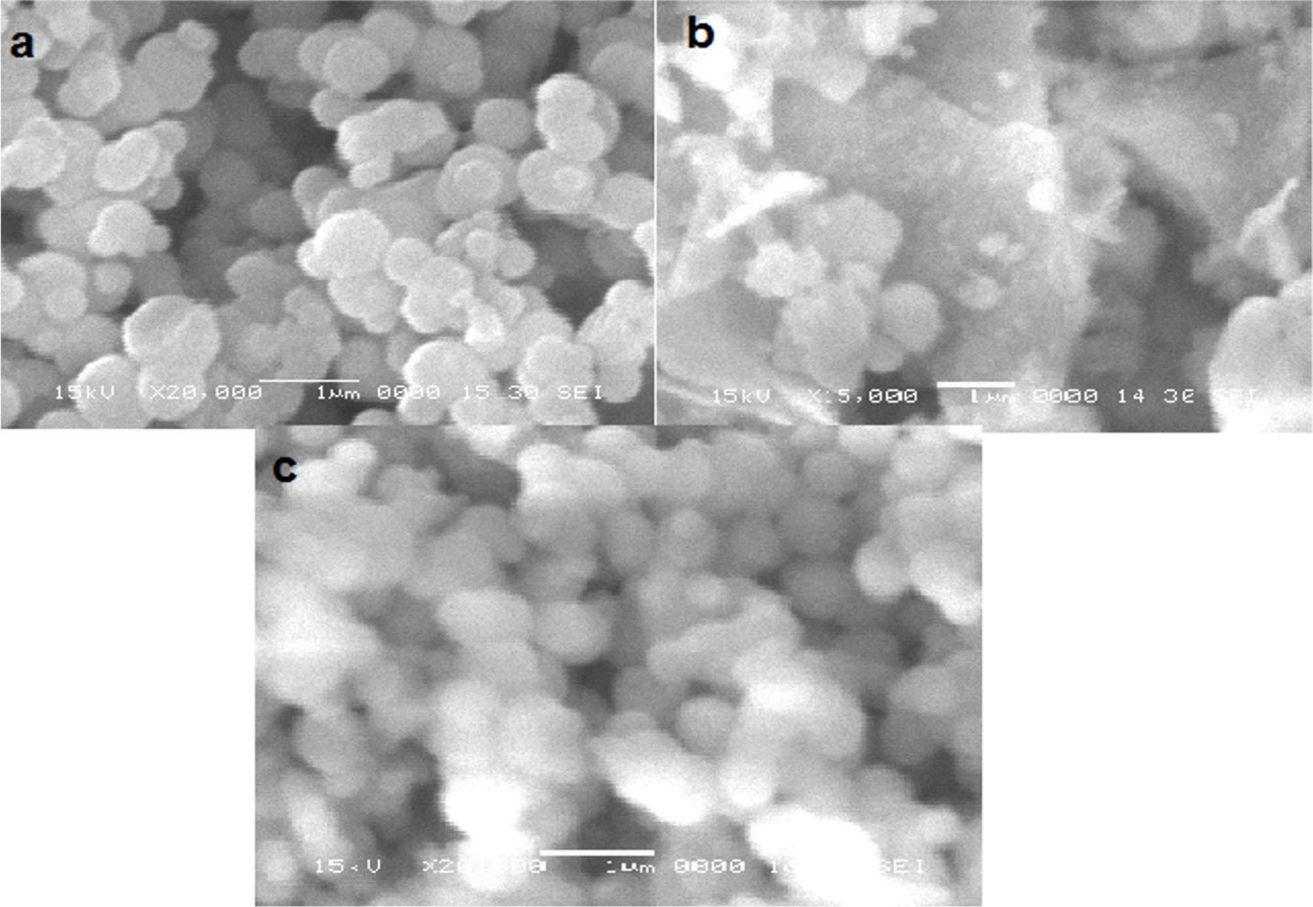
Scanning Electron Microscopy (SEM) of: (**a**) mesoporous silica NPs by homogenous system; (**b**) mesoporous silica NPs by heterogenous system; and (**c**) MCM-48 NPs

**Fig. 4:**
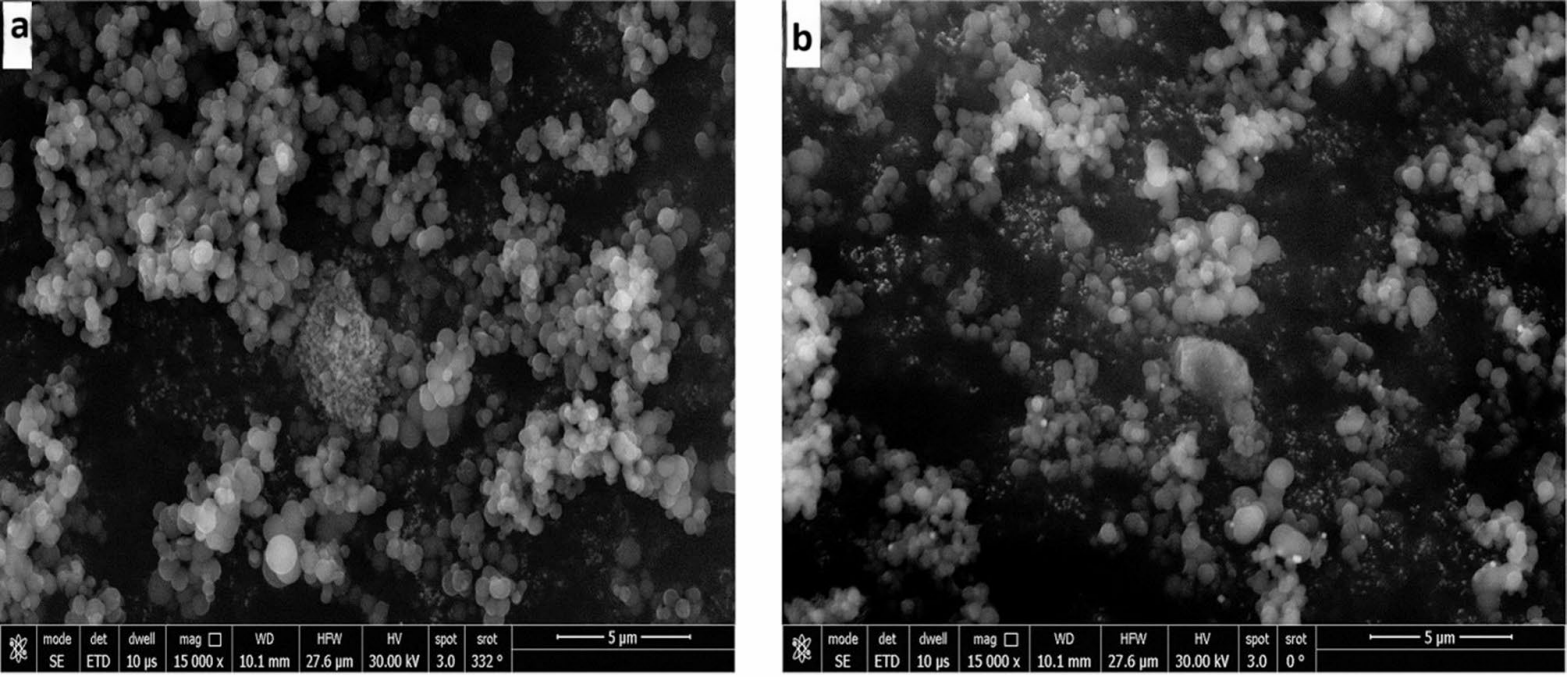
Scanning Electron Microscopy (SEM) of: (**a**) Ag/Zn/MCM-48 NPs (10 mmol); and (**b**) Ag/MCM-48 (10 mmol) NPs

The surface morphology of different types of mesoporous silica and silver/zinc incorporated MCM-48 NPs was obtained by SEM, and the spherical shape of MCM-48 is shown in Fig. 3. The mean size of MSN synthesized by a homogenous system was obtained as 469.4 nm. The MSN synthesized by a heterogeneous system mean size was found to be 559.4 nm with high degree of agglomeration. The SEM of MCM-48 NPs demonstrated a mean size of 413.5 nm with no aggregates. The MCM-48 NPs and MSN by the homogenous system showed off spherical shape, while Ag/MCM-48 NPs (10 mmol) showed spherical particles with a mean size equal to 481 nm with no aggregates, while Ag/Zn/MCM-48 NPs (10 mmol) presented a mean size equal to 450.9 nm with no aggregates (Fig. 4). This verified the successful synthesis, loading of the metals, and the homogeneity of the NPs after the introduction of the metals on MCM-48 NPs.

### Antibacterial activities

As displayed in Table 1, the Ag/MCM-48 NPs (10 mmol) and Ag/Zn/MCM-48 NPs (10 mmol) displayed antibacterial effects measured by the zones of inhibition of 16.3 mm and 9.6 mm against *S. aureus* ATCC 25,923 and 17.3 mm and 10.3 mm against *E. coli* ATCC 25,922, respectively. On the other hand, MCM-48 NPs alone did not show any antibacterial effect (no zone of inhibition was detected) against either reference strains or MDR isolates (Table 1). The Ag/MCM-48 NPs (10 mmol) and Ag/Zn/MCM-48 NPs showed remarkable inhibition zones ranging from 16 to 35 mm and 14–40 mm against the tested MDR pathogens, respectively (Tables 2, and 3). The recorded zone of inhibition zones of Ag/MCM-48 NPs and Ag/Zn/MCM-48 NPs against the tested bacterial isolates are depicted in Figs. S2-S9 (supplementary file). MIC of the Ag/MCM-48 (10 mmol) and Ag/Zn/MCM-48 (10 mmol) NPs ranged from 7.8 to 31.25 µg/mL against the tested MDR bacterial pathogens, confirming their remarkable antibacterial activities (Table 4**).**

**Table 2:** Antibacterial activities of the MCM-48 nps, Ag/MCM-48 (10 mmol), and Ag/Zn/MCM-48 NPs (10 mmol) against two reference strains

Reference strains	Average inhibition zone diameter (mm) ± SD			
	MCM-48 NPs	Ag/MCM-48 (10 mmol)	Ag/Zn/MCM-48 NPs (10 mmol)	Gentamicin (4 µg/mL)
*S. aureus* ATCC 25,923	0	16.3 ± 0.66	9.6 ± 0.66	24.3 ± 1.33
*E. coli* ATCC 25,922	0	17.3 ± 0.66	10.3 ± 0.66	30.3 ± 0.66

**Table 3:** Antibacterial activities of the MCM-48 nps, Ag/MCM-48 (10 mmol), and Ag/Zn/MCM-48 NPs (10 mmol) against six MDR isolates

Reference strains/MDR isolates	Average inhibition zone diameter (mm) ± SD				
	MCM-48 NPs	Ag/MCM-48 (10 mmol)	Ag/Zn/MCM-48 NPs (10 mmol)	Gentamicin (4 µg/mL)	Ciprofloxacin (10 µg/mL)
MDR *E. coli* 48 WE	0	16.6 ± 1.33	14.3 ± 1.33	16.6 ± 0.66	NT
MDR *K. pneumoniae* 36 AK	0	19.3 ± 0.66	15.6 ± 0.66	ND	15.3 ± 1.33
MRSA ATCC 33,591	0	29.3 ± 1.33	23.6 ± 0.33	ND	25.6 ± 0.33
MDR *E. coli* 25 LN	0	35.6 ± 0.66	40.3 ± 0.66	ND	31.3 ± 0.66
MDR *K. pneumoniae* 78 TE	0	25.3 ± 1.33	28.6 ± 1.33	ND	26.3 ± 1.33
MDR *Salmonella* Typhi 24 ZA	0	26.3 ± 0.33	24.3 ± 1.33	ND	25.3 ± 1.33

NT not tested; ND, not determined as the tested isolates were resistant; MRSA, methicillin-resistant *S. aureus*; MDR, multidrug-resistant; MCM-48 NPs, mobile composition of matter no. 48 nanoparticles

**Table 4:** MIC values of the Ag/MCM-48 (10 mmol) and Ag/Zn/MCM-48 NPs (10 mmol) against four MDR pathogens

Reference strains/MDR isolates	MIC measurement (µg/mL)			
	Ag/MCM-48 (10 mmol)	Ag/Zn/MCM-48 NPs (10 mmol)	Gentamicin	Ciprofloxacin
MRSA ATCC 33,591	15.62	31.25	ND	0.5
MDR *E. coli* 25 LN	7.8	7.8	ND	1.0
MDR *K. pneumoniae* 78 TE	31.25	31.25	ND	1.0
MDR *Salmonella* Typhi 24 ZA	31.25	31.25	ND	2.0

	**MBC measurement (µg/mL)**			
MRSA ATCC 33591	31.25	31.25		
MDR* E. coli *25 LN	15.62	7.8		
MDR* K. pneumoniae *78 TE	62.5	31.25		
MDR* Salmonella *Typhi24 ZA	31.25	31.25		

### In vitro antibiofilm activity

**Fig. 5:**
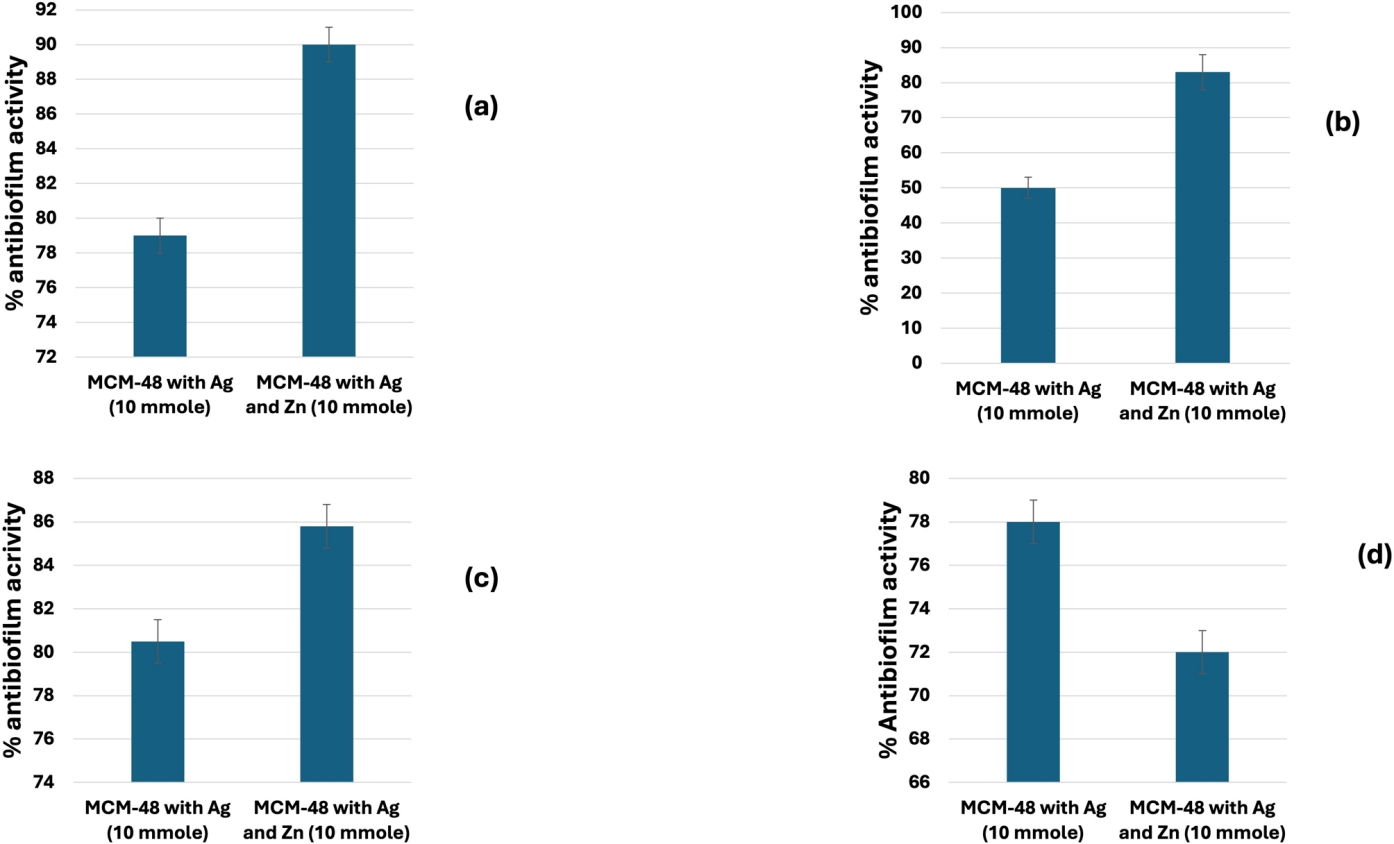
Antibiofilm activity (%reduction in the biofilm formation) of Ag/MCM-48 and Ag/Zn/MCM-48 against the biofilm formed (100%) b : (**a**) *E. coli* 25 LN; (**b**) *K. pneumoniae* 78 TE; (**c**) *Salmonella* Typhi 24 ZA; and (**d**) MRSA ATCC 33,591

As depicted in Fig. 5, the Ag/Zn/MCM-48 nanoparticles displayed higher % reduction in the formed biofilm (antibiofilm activity) of about 90%, 83%, and 86% against *E. coli* 25 LN (Fig. 5a), *K. pneumoniae* 78 TE (Fig. 5b), and *Salmonella* Typhi 24 ZA (Fig. 5c) as compared to Ag/MCM-48 nanoparticles, respectively. On the other hand, the Ag/MCM-48 nanoparticles exhibited higher antibiofilm activity (78%) compared to Ag/Zn/MCM-48 (71%) against MRSA only (Fig. 5d).

### Cytotoxic activities

**Fig. 6:**
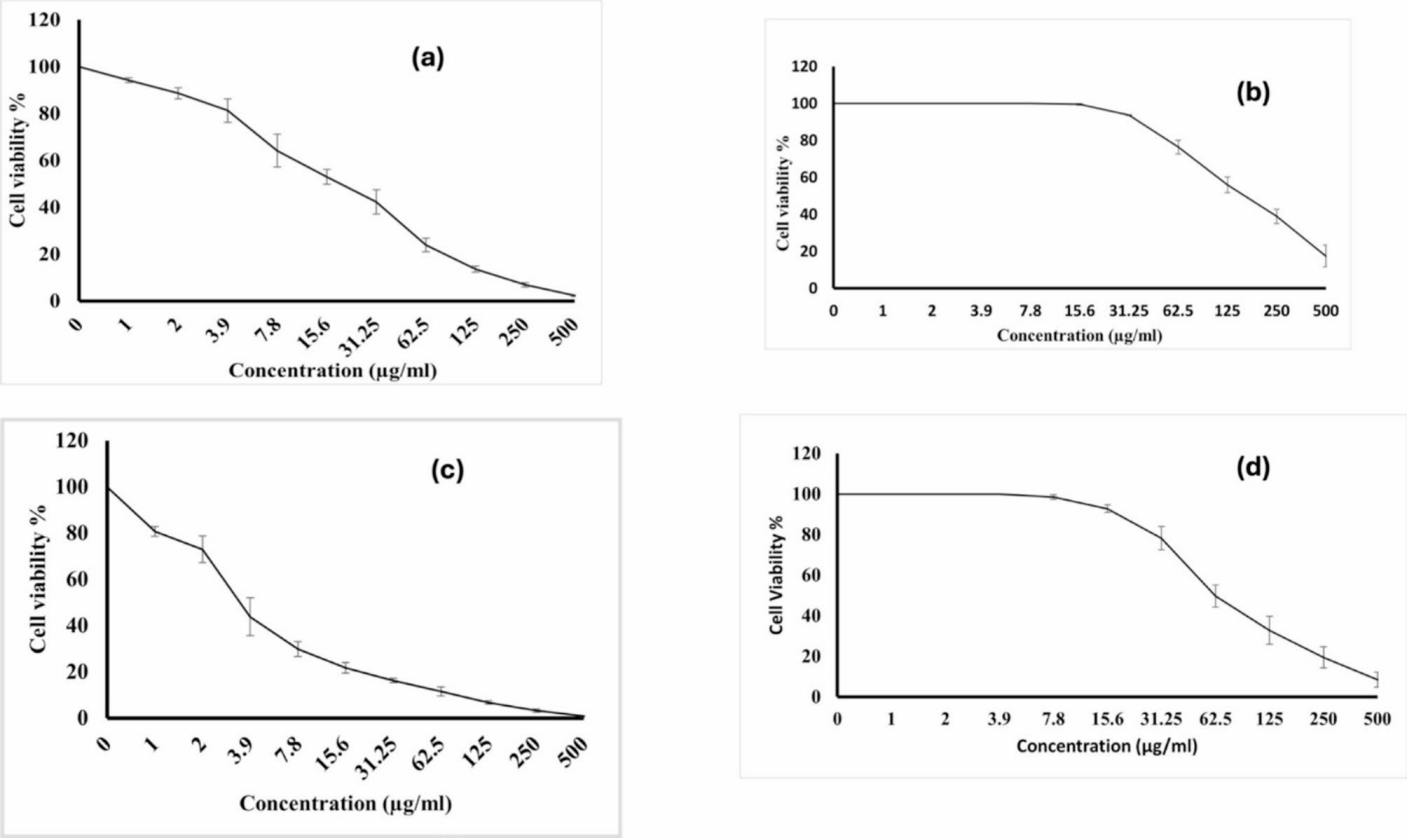
The relation between % cell viability versus concentration of nanoparticles: (**a**) Ag/MCM-48 NPs (10 mmol) against *Vero* cell line; (**b**) Ag/Zn/MCM-48 NPs (10 mmol) against *Vero* cell line; (**c**) Ag/MCM-48 NPs (10 mmol) against *Caco*−2 cell line; (**d**) Ag/Zn/MCM-48 NPs (10 mmol) against *Caco*−2 line

As shown in Fig. 6, the cytotoxicity of Ag/MCM-48 NPs (10 mmol) on the *Vero* cell line was measured, yielding the cytotoxic concentration of test sample needed to lessen cell viability by 50% (CC_50_) of 19.95 ± 0.63 µg/mL (Fig. 6a). In contrast, the cytotoxic activity of Ag/Zn/MCM-48 NPs (10 mmol) against the same cell line resulted in a CC_50_ of 169.16 ± 6.43 µg/mL (Fig. 6b). The synthesized Ag/MCM-48 NPs (10 mmol) exhibited higher cytotoxicity than the Ag/Zn/MCM-48 NPs (10 mmol) on *Caco*−2 with an inhibition concentration of test sample that inhibits 50% of biological function (IC_50_) = 3.58 0.12 µg/mL (Fig. 6c), while Ag/Zn/MCM-48 NPs (10 mmol) showed an IC_50_ of 62.17 ± 2.15 µg/mL (Fig. 6d). The percentage inhibitory and percentage viability of the tested cell lines using different concentrations of Ag/MCM-48 NPs (10 mmol) and Ag/Zn/MCM-48 NPs (10 mmol) nanoparticles are displayed in Tables S1-S4 (supplementary data).

## Discussion

Research has pointed out that silver nanoparticles exhibit significant antibacterial activity against Gram-positive and Gram-negative bacteria through diverse mechanisms of action (Altammar 2023). However, their cytotoxicity remains a major limitation (Abass Sofi et al. 2022). In this work, silver nanoparticles’ cytotoxicity was investigated using an innovative approach. First, MSNs were synthesized to act as carriers. MSNs were synthesized by three different methods to obtain the smallest particle size MSNs with high yield to continue with: MSNs by a homogenous system with CTAB, MSNs by a heterogeneous system, and MCM-48 NPs. MCM-48 NPs exhibited the smallest particle size among the three MSNs, which provided a higher surface area for metals incorporation, which was achieved by MCM-48 NPs with a grain size of 101.01 nm, with spherical shape. Two variants of mesoporous silica nanoparticles were synthesized: Ag/MCM-48 NPs (10 mmol Ag) and Ag/Zn/MCM-48 NPs (10 mmol Ag + 10 mmol Zn). They were successfully synthesized and confirmed by XRD and SEM analysis. Secondly, antibacterial tests were done on different isolates, revealing that MCM-48 NPs showed no antibacterial activity against *S. aureus* ATCC 25,923, *E. coli* ATCC 25,922, *E. coli* 48 WE, *K. pneumoniae* 36 AK, and *Salmonella* Typhi 24 ZA, consistent with Jin et al., as it is predicted that MSNs have no antibacterial effect on a wide range of pathogens and are mainly used in combinations with other antimicrobial agents (Jin et al. 2023). Conversely, the incorporation of Ag (10 mmol) to MCM-48 (Ag/MCM-48 NPs) resulted in clear inhibition zones against all the tested isolates, measuring between 16 mm and 35 mm, affirming the good antibacterial activity of silver stated by previous literature (He et al. 2022). Ag/MCM-48 showed slightly bigger inhibition zone against *E. coli* ATCC 25,922 and other *S. aureus* strains than Ag NPs/MCM-41 which was synthesized by (Asli et al. 2022). MSNs acted as carriers for silver and adhered to bacterial cells, facilitating release of silver ions, which act as follows: (1) These ions interact with the negative charges on the bacterial cell membrane. (2) Interact with sulfur-containing proteins. (3) Stopping DNA replication. (4) Causing cell death (Dhaka et al. 2023; Salleh et al. 2020; Salmani-Zarchi et al. 2024). The formulated Ag/Zn/MCM-48 NPs (10 mmol) also presented clear inhibition zones against all the bacterial isolates, ranging from 9 mm to 40 mm. However, their antibacterial activity against *S. aureus* ATCC 25,923, *E. coli* ATCC 25,922, *E. coli* 48 WE, *K. pneumoniae* 36 AK, *S.* Typhi 24 ZA and MRSA ATCC 33,591 was slightly less potent than Ag/MCM-48 NPs (10 mmol). In contrast, *E. coli* 25 LN and *K. pneumoniae* 78 TE showed greater sensitivity to Ag/Zn/MCM-48 NPs (10 mmol) compared to Ag/MCM-48 NPs (10 mmol). The MIC test was conducted to further investigate the synthesized nanoparticles’ antibacterial effect. Results revealed that Ag/MCM-48 NPs (10 mmol) had a lower MIC of 15.62 µg/mL against MRSA ATCC 33,591 compared to 31.25 µg/mL of Ag/Zn/MCM-48 NPs (10 mmol). Both nanoparticle samples had the same MIC of 7.8 µg/mL against *E. coli* 25 LN, and 31.25 µg/mL against *K. pneumoniae* 78 TE and 31.25 µg/mL against *S.* Typhi 24 ZA. It is evident that Ag/MCM-48 NPs (10 mmol) and Ag/Zn/MCM-48 NPs (10 mmol) completely inhibited the growth of all four bacteria at only 31.25 µg/mL, demonstrating comparable antibacterial activity.

The in vitro biofilm formation was assessed using half the MIC of the prepared nanoparticles. The Ag/Zn/MCM-48 NPs (10 mmol) showed higher antibiofilm activity, achieving 90% reduction compared to 79% for Ag/MCM-48 NPs (10 mmol) against *E. coli* 25 LN. Additionally, Ag/Zn/MCM-48 NPs (10 mmol) had greater antibiofilm activity with reductions of 85% and 84% against *S.* Typhi 24 ZA and *K. pneumoniae* 78 TE, respectively. This contrasted with Ag/MCM-48 NPs (10 mmol), which showed reductions of 80% and 51%. However, Ag/MCM-48 (10 mmol) NPs represented slightly higher antibiofilm activity against MRSA ATCC 33,591 with a reduction of 78% compared to 72% for Ag/Zn/MCM-48 NPs (10 mmol). In comparison with recent literature, silver showed better antibiofilm activity against *E. coli* than zinc, in contrast with our work, while showing greater antibiofilm activity than zinc against *Staphylococcus aureus*, which aligns with our results (Lekhan et al. 2022). The enhanced antibiofilm activity of Ag/Zn/MCM-48 NPs (10 mmol) may be attributed to the binding of silver and zinc NPs to the extracellular polymeric substance on the bacterial cells. Once connected to a biofilm, silver and zinc NPs interact with a complex mix of macromolecules, causing variations in their surface features and creating a biomolecular corona (Sarkar et al. 2024). Zinc ions, as well as ROS production, bind to zinc import channels, bonding with Mn importers, causing Mn starvation followed by respiration damage to bacterial cells. With silver ions damaging the cells as mentioned previously, causing the synergistic effect of both silver and zinc (Es-haghi et al.^2024^; Madipoju et al. 2025; Makauki et al. 2025).

Silver nanoparticles produce significant reactive oxidative stress (ROS) that can destroy the cell membrane and elevate the risk of cytotoxicity (Nie et al. 2023). To overcome this challenge, silver nanoparticles were incorporated with zinc nanoparticles into the MCM-48 network. The in vitro cytotoxicity effect of the synthesized nanoparticles was evaluated on the *Caco*−2 cell line and the *Vero* cell line using the MTT assay. The synthesized Ag/MCM-48 NPs (10 mmol) exhibited lower cell viability compared to Ag/Zn/MCM-48 NPs (10 mmol) on the *Vero c*ell line, with a CC_50_ of 19.95 ± 0.63 µg/mL for Ag/MCM-48 NPs and 169.16 ± 6.43 µg/mL for Ag/Zn/MCM-48 NPs. Concerning cytotoxicity on the *Caco*−2 cell line, the Ag/MCM-48 NPs (10 mmol) showed greater cytotoxicity than the synthesized Ag/Zn/MCM-48 NPs (10 mmol). The IC_50_ was found to be 3.58 ± 0.12 µg/mL for Ag/MCM-48 NPs, while Ag/Zn/MCM-48 NPs (10 mmol) had an IC_50_ of 62.17 ± 2.15 µg/mL. This indicated that Ag/MCM-48 nanoparticles are more harmful to *Caco*−2 cells, whereas Ag/Zn/MCM-48 nanoparticles are safe and less toxic to both cell lines. Accordingly, Ag/Zn/MCM-48 NPs are more toxic to cancerous cell lines than the normal cell line, which is hopeful in cancer treatment with less side effects in agreement with a previous study (Helalat et al. 2023).

These results may be attributed to the fact that zinc enhances cell proliferation as it acts as a cofactor for more than 300 enzymes that participate in cell division (Zhang et al. 2023) and proves that zinc and silver combination reduces cytotoxicity with a favorable antibacterial effect, in agreement with Leng et al. Furthermore, zinc participates in homeostasis. The presence of zinc in cell is distributed into three locations: (1) Zn^2^ conjoined proteins. (2) Unbound Zn^2^ in cytoplasm. (3) Zn^2^ in organelles. Intracellular zinc concentration is maintained by Zn-transporters (Liu et al. 2023). Balanced zinc levels: help gene expression transcription and translation, facilitate the maturation and regulation of immune cells such as T-lymphocytes (Yao et al. 2025), play an important role in insulin synthesis, storage, and secretion (Fukunaka and Fujitani 2018; Leng et al. 2020). Zinc is well known for its inhibitory effect on caspase-3 (cysteine-aspartic proteases), which affects regulated cell death and apoptosis (Briassoulis et al. 2023).

In this work, definite concentrations of silver-incorporated mesoporous silica nanoparticles and silver/zinc-incorporated mesoporous silica nanoparticles were synthesized and showed promising antimicrobial activity. Their physicochemical characterization confirmed successful synthesis, demonstrating that the synthesized nanoparticles were homogenous, confirming the presence of silver and zinc within the MCM-48 network. The in vitro antibacterial assays conducted on the synthesized nanoparticle samples showed potential results against MRSA, *K. pneumoniae* 78 TE, *E. coli* 25 LN, and *S.* Typhi 24 ZA. While Ag/MCM-48 exhibited cytotoxic activity towards *Vero* and *Caco-2* cell lines, Ag/Zn/MCM-48 NPs proved to be significantly safer. This could be attributed to the synergistic effect of combining silver and zinc, which enhances their antimicrobial properties and reduces cytotoxic activity, suggesting a promising application as potential anticancer agents. The results highlighted the strong potential of these prepared nanoparticles as targeted drug delivery and as antimicrobial agents against MDR organisms. The fact that mesoporous silica NPs were used as carriers is advantageous in preventing aggregation and enhancing cell targeting to reduce the cytotoxic effect. Furthermore, other drugs such as antibiotics and anticancer drugs could also be carried on mesoporous silica NPs, allowing their selective and targeted delivery to cells.

## Supplementary Information

Supplementary Material 1

## Data Availability

All data generated or analyzed during this study are included in this published article and supplementary file.

## Abbreviations

MTT: (3-(4,5-dimethylthiazol-2-yl)-2,5-diphenyltetrazolium bromide)
HEPES: (4-(2-hydroxyethyl)-1-piperazineethanesulfonic acid) buffer solution
DMEM: (Dulbecco’s modified Eagle’s medium)
Caco-2: (intestinal carcinoma cell line)
Vero: (Mammalian cells from African Green Monkey Kidney)
RPMI-1640: (Roswell Park Memorial Institute)
AMR: Antimicrobial resistance
CDC: Center for Disease Control and Prevention
CTAB: Cetyltrimethylammonium bromide
DMSO: Dimethyl sulfoxide
GRAS: Generally recognized as safe
IC50: Inhibition concentration of test sample that inhibits 50% of biological function
JCPDS: Joint Committee on Powder Diffraction Standards
MSN: Mesoporous silica nanoparticles
MRSA: Methicillin-resistant*Staphylococcus aureus*
MBC: Minimum bactericidal concentrations
MIC: Minimum inhibitory concentrations
MDR: Multidrug-resistant pathogens
PBP-2A: Penicillin binding protein
ROS: Reactive Oxygen species
SEM: Scanning electron microscopy
AgNPs: Silver nanoparticles
TEOS: Tetraethyl orthosilicate
NPs: that nanoparticles
CCASU: the Ain Shams University culture collection
ATCC: the American Type Culture Collection
CLSI: the Clinical & Laboratory Standards Institute
CC50: the cytotoxic concentration of test sample needed to lessen cell viability by 50%
RCMB: the Egypt Regional Center for Mycology and Biotechnology
TSB: Tryptic soy broth
WHO: World Health Organization
XRD: X-ray diffraction

## References

[CR1] Abass Sofi M, Sunitha S, Ashaq Sofi M, Khadheer Pasha SK, Choi D (2022) An overview of antimicrobial and anticancer potential of silver nanoparticles. J King Saud Univ 34:101791. 10.1016/j.jksus.2021.101791

[CR2] Abuzeyad OH, El-Khawaga AM, Tantawy H, Elsayed MA (2023) An evaluation of the improved catalytic performance of rGO/GO-hybrid-nanomaterials in photocatalytic degradation and antibacterial activity processes for wastewater treatment: a review. J Mol Struct 1288:1–15. 10.1016/j.molstruc.2023.135787

[CR3] Abuzeyad OH, El-Khawaga AM, Tantawy H, Gobara M, Elsayed MA (2025) Reduced graphene oxide loaded with ZCF magnetic nanoparticles as a promising photocatalyst and antibacterial agent. J Clust Sci. 10.1007/s10876-024-02718-6

[CR4] Ahmed MS, Hussein NN, Sulaiman GM, Khan RA, Mohammed HA (2025) Piperacillin-loaded amine functionalized mesoporous silica nanoparticles: a new frontier in combating multidrug-resistant pathogenic bacteria through value-added Piperacillin antibiotic. J Drug Deliv Sci Technol. 10.1016/j.jddst.2024.106580

[CR5] Al-Momani H, Massadeh MI, Almasri M, Al Balawi D, Aolymat I, Hamed S, Albiss BA, Ibrahim L, Balawi H, Al S (2024) Anti-Bacterial Activity of Green Synthesised Silver and Zinc Oxide Nanoparticles against Propionibacterium acnes. Pharmaceuticals 17:255. 10.3390/ph17020255 10.3390/ph17020255 PMC10891609 38399471

[CR6] Aloke C, Egwu CO, Onisuru OO, Otun S, Achilonu I (2025) Exploiting the therapeutic efficacy of nanoparticles in the treatment of multidrug-resistant bacteria: excitements and pitfalls. J Drug Deliv Sci Technol 104:106501. 10.1016/j.jddst.2024.106501

[CR7] Altammar KA (2023) A review on nanoparticles: characteristics, synthesis, applications, and challenges. Front Microbiol. 10.3389/fmicb.2023.1155622 37180257 10.3389/fmicb.2023.1155622 PMC10168541

[CR8] Asli B, Abdelkrim S, Zahraoui M, Mokhtar A, Hachemaoui M, Bennabi F, Ahmed AB, Sardi A, Boukoussa B (2022) Catalytic reduction and antibacterial activity of MCM-41 modified by silver nanoparticles. Silicon 14:12587–12598. 10.1007/s12633-022-01963-6

[CR9] Balouiri M, Sadiki M, Ibnsouda SK (2016) Methods for in vitro evaluating antimicrobial activity: a review. J Pharm Anal 6:71–79. 10.1016/j.jpha.2015.11.005 29403965 10.1016/j.jpha.2015.11.005 PMC5762448

[CR10] Banoub NG, Saleh SE, Helal HS, Aboshanab KM (2021) Antibiotics combinations and chitosan nanoparticles for combating multidrug resistance *Acinetobacter baumannii*. Infect Drug Resist 14:3327–3339. 10.2147/idr.s328788 34447258 10.2147/IDR.S328788 PMC8384262

[CR11] Basavegowda N, Baek K-H (2021) Multimetallic nanoparticles as alternative antimicrobial agents: challenges and perspectives. Molecules 26:912. 10.3390/molecules26040912 33572219 10.3390/molecules26040912 PMC7915418

[CR12] Bidaki MZ, Naghizadeh A, Yousefinia A, Hosseinzadeh M, Lashkari S, Mortazavi-Derazkola S, Moghanni M (2025) Environmentally friendly synthesis of silver nanoparticles using prickly pear extract and their antimicrobial and antioxidant activities. Biomass Convers Biorefin 15:3631–3640. 10.1007/s13399-023-05259-6

[CR13] Briassoulis G, Briassoulis P, Ilia S, Miliaraki M, Briassouli E (2023) The anti-oxidative, anti-inflammatory, anti-apoptotic, and anti-necroptotic role of zinc in COVID-19 and sepsis. Antioxidants 12(11):1942. 10.3390/antiox12111942 38001795 10.3390/antiox12111942 PMC10669546

[CR14] Chen L, Kumar S, Wu H (2023) A review of current antibiotic resistance and promising antibiotics with novel modes of action to combat antibiotic resistance. Arch Microbiol 205(11):356. 10.1007/s00203-023-03699-2 37863957 10.1007/s00203-023-03699-2

[CR15] Church NA, Mckillip JL (2021) Antibiotic resistance crisis: challenges and imperatives. Biologia 76, 1535–1550 10.1007/s11756-021-00697-x

[CR16] CLSI (2021) CLSI: Performance standards for antimicrobial susceptibility testing. Clinical and Laboratory Standards Institute 2021. In: vol. M100-Ed31

[CR17] Czyżowska A, Barbasz A (2022) A review: zinc oxide nanoparticles – friends or enemies? Int J Environ Health Res 32:885–901. 10.1080/09603123.2020.1805415 32772735 10.1080/09603123.2020.1805415

[CR18] da Rosa TF, Coelho SS, Foletto VS, Bottega A, Serafin MB, Machado C, de Franco S, de Paula LN, Hörner BR R (2020) Alternatives for the treatment of infections caused by ESKAPE pathogens. J Clin Pharm Ther 45(4):863–873. 10.1111/jcpt.13149 32339305 10.1111/jcpt.13149

[CR19] Dhaka A, Chand Mali S, Sharma S, Trivedi R (2023) A review on biological synthesis of silver nanoparticles and their potential applications. Results Chem 6:101108. 10.1016/j.rechem.2023.101108

[CR20] EClinicalMedicine (2021) Antimicrobial resistance: a top ten global public health threat. EClinicalMedicine 41:101221. 10.1016/j.eclinm.2021.101221 34877512 10.1016/j.eclinm.2021.101221 PMC8633964

[CR21] El-Khawaga AM, Tantawy H, Elsayed MA, Abd El-Mageed AIA (2022) Synthesis and applicability of reduced graphene oxide/porphyrin nanocomposite as photocatalyst for waste water treatment and medical applications. Sci Rep. 10.1038/s41598-022-21360-8 36224230 10.1038/s41598-022-21360-8 PMC9556635

[CR22] El-Saadony M, Fang G, Yan S, Alkafaas S, El Nasharty M, Khedr S, Hussien A, Ghosh S, Dladla M, Elkafas SS, Ibrahim E, Salem H, Mosa W, Ahmed A, Mohammed DM, Korma S, El-Tarabily M, Saad A, El-Tarabily K, AbuQamar S (2024) Green synthesis of zinc oxide nanoparticles: preparation, characterization, and biomedical Applications - A review. Int J Nanomed Volume 19:12889–12937. 10.2147/IJN.S487188 10.2147/IJN.S487188 PMC11624689 39651353

[CR23] Es-haghi A, Amiri MS, Taghavizadeh Yazdi ME (2024) *Ferula latisecta* gels for synthesis of zinc/silver binary nanoparticles: antibacterial effects against gram-negative and gram-positive bacteria and physicochemical characteristics. BMC Biotechnol. 10.1186/s12896-024-00878-x 39090578 10.1186/s12896-024-00878-x PMC11292920

[CR25] Fahim YA, El-Khawaga AM, Sallam RM, Elsayed MA, Assar MFA (2024) Immobilized lipase enzyme on green synthesized magnetic nanoparticles using *psidium guava* leaves for dye degradation and antimicrobial activities. Sci Rep. 10.1038/s41598-024-58840-y 38627424 10.1038/s41598-024-58840-y PMC11021406

[CR26] Fukunaka A, Fujitani Y (2018) Role of zinc homeostasis in the pathogenesis of diabetes and obesity. Int J Mol Sci 19(2):476. 10.3390/ijms19020476 29415457 10.3390/ijms19020476 PMC5855698

[CR27] Hamad A, Khashan KS, Hadi A (2020) Silver nanoparticles and silver ions as potential antibacterial agents. J Inorg Organomet Polym Mater 30:4811–4828. 10.1007/s10904-020-01744-x

[CR28] He X, Chen F, Chang Z, Waqar K, Hu H, Zheng X, Wang Y, Dong W, fei, Yang C (2022) Silver mesoporous silica nanoparticles: fabrication to combination therapies for cancer and infection. Chem Rec 22(4):e202100287. 10.1002/tcr.202100287 35020240 10.1002/tcr.202100287

[CR29] Helalat R, Masoumeh MO, Rezaei N, Baghbani-Arani F (2023) Evaluation of the cytotoxic effects of silver-zinc oxide nanoparticles synthesized by green method on sw480 cell line. Emergent Mater 6:291–298. 10.1007/s42247-022-00413-8

[CR30] Hochvaldová L, Večeřová R, Kolář M, Prucek R, Kvítek L, Lapčík L, Panáček A (2022) Antibacterial nanomaterials: upcoming hope to overcome antibiotic resistance crisis. Nanotechnol Rev 11:1115–1142

[CR31] Horie M, Tabei Y (2021) Role of oxidative stress in nanoparticles toxicity. Free Radic Res 55:331–342. 10.1080/10715762.2020.1859108 33336617 10.1080/10715762.2020.1859108

[CR32] Hoseini-Alfatemi SM, Karimi A, Armin S, Fakharzadeh S, Fallah F, Kalanaky S (2018) Antibacterial and antibiofilm activity of nanochelating based silver nanoparticles against several nosocomial pathogens. Appl Organomet Chem. 10.1002/aoc.4327

[CR33] Hossain ML, Lim LY, Hammer K, Hettiarachchi D, Locher C (2022) A review of commonly used methodologies for assessing the antibacterial activity of honey and honey products. Antibiotics 11:975. 10.3390/antibiotics11070975 35884229 10.3390/antibiotics11070975 PMC9312033

[CR34] Hudaya IR, Situmorang VC, Hardianto FA, Putra EDL, Satria D, Hertriani T, Hartati R, Septama AW (2024) Biofabrication of silver nanoparticles using *Artocarpus heterophyllus* leaves extract: characterization and evaluation of its antibacterial, antibiofilm, and antioxidant activities. J Cryst Growth 644:127827. 10.1016/j.jcrysgro.2024.127827

[CR35] Jahan Tamanna N, Sahadat Hossain M, Mohammed Bahadur N, Ahmed S (2024) Green synthesis of Ag2O & facile synthesis of ZnO and characterization using FTIR, bandgap energy & XRD (Scherrer equation, Williamson-Hall, size-train plot, Monshi- Scherrer model). Results Chem 7. 10.1016/j.rechem.2024.101313

[CR36] Jin Y, Lu Y, Sathiyaseelan A, Saravanakumar K, Zhang X, Wang MH (2023) Characterization, cytotoxicity, and antibacterial activity of paeoniflorin-loaded mesoporous silica oxide nanoparticles. J Drug Deliv Sci Technol. 10.1016/j.jddst.2023.104551

[CR37] Kalita A, Kashyap T, Saikia P, Talukdar AK (2024) In-situ iron modified mesoporous silica MCM-48 for electrochemical energy storage applications. J Porous Mater 31:2067–2082. 10.1007/s10934-024-01657-x

[CR38] Kim I, Viswanathan K, Kasi G, Thanakkasaranee S, Sadeghi K, Seo J (2022) *ZnO* nanostructures in active antibacterial food packaging: preparation methods, antimicrobial mechanisms, safety issues, future prospects, and challenges. Food Rev Int 38:537–565. 10.1080/87559129.2020.1737709

[CR39] Kumar D, Schumacher K, du, von Hohenesche F, Grün C, Unger M (2001) KK MCM-41, MCM-48 and related mesoporous adsorbents: their synthesis and characterisation. Colloids Surf A Physicochem Eng Asp 187–188:109–116. 10.1016/S0927-7757(01)00638-0

[CR40] Kuznetsova MV, Nesterova LY, Mihailovskaya VS, Selivanova PA, Kochergina DA, Karipova MO, Valtsifer IV, Averkina AS, Starčič Erjavec M (2025) Nosocomial *Escherichia coli*, *Klebsiella pneumoniae*, *Pseudomonas aeruginosa*, and *Staphylococcus aureus*: sensitivity to chlorhexidine-based biocides and prevalence of efflux pump genes. Int J Mol Sci 26:355. 10.3390/ijms26010355 39796210 10.3390/ijms26010355 PMC11721292

[CR41] Lekhan A, Fiore C, Shemchuk O, Grepioni F, Braga D, Turner RJ (2022) Comparison of antimicrobial and antibiofilm activity of proflavine co-crystallized with silver, copper, zinc, and gallium salts. ACS Appl Bio Mater 5:4203–4212. 10.1021/acsabm.2c00404 35970511 10.1021/acsabm.2c00404 PMC9491326

[CR42] Leng D, Li Y, Zhu J, Liang R, Zhang C, Zhou Y, Li M, Wang Y, Rong D, Wu D, Li J (2020) ) < p > the antibiofilm activity and mechanism of Nanosilver- and Nanozinc-Incorporated mesoporous Calcium-Silicate nanoparticles. Int J Nanomed Volume 15:3921–3936. 10.2147/IJN.S244686. 10.2147/IJN.S244686 PMC7278446 32581537

[CR43] Liu J, Li S, Fang Y, Zhu Z (2019) Boosting antibacterial activity with mesoporous silica nanoparticles supported silver nanoclusters. J Colloid Interface Sci 555:470–479. 10.1016/j.jcis.2019.08.009 31400539 10.1016/j.jcis.2019.08.009

[CR44] Liu S, Wang N, Long Y, Wu Z, Zhou S (2023) Zinc homeostasis: an emerging therapeutic target for neuroinflammation related diseases. Biomolecules 13(3):416. 10.3390/biom13030416 36979351 10.3390/biom13030416 PMC10046788

[CR45] Madipoju NK, Khanam SJ, Sunkari G, Vasamsetti SM, Divyasri V, Morampudi V, Savu RN, Banavoth M, Banne S, Hasan I, Kalakonda P (2025) Bimetallic zinc-silver nanocomposites for superior antimicrobial efficacy. Appl Phys A 131:153. 10.1007/s00339-025-08289-1

[CR46] Magaldi S, Mata-Essayag S, Hartung de Capriles C, Perez C, Colella MT, Olaizola C, Ontiveros Y (2004) Well diffusion for antifungal susceptibility testing. Int J Infect Dis 8:39–45. 10.1016/j.ijid.2003.03.002 14690779 10.1016/j.ijid.2003.03.002

[CR47] Makauki E, Machunda R, Basu O, Rwiza M (2025) Synergistic antimicrobial mechanisms of silver-doped zinc oxide for water treatment: a systematic review. H2Open J 8:72–88. 10.2166/h2oj.2025.037

[CR48] Manzano M, Vallet-Regí M (2020) Mesoporous silica nanoparticles for drug delivery. Adv Funct Mater. 10.1002/adfm.201902634

[CR49] Mohamed Isa ED, Ahmad H, Abdul Rahman MB, Gill MR (2021) Progress in mesoporous silica nanoparticles as drug delivery agents for cancer treatment. Pharmaceutics 13:152. 10.3390/pharmaceutics13020152 33498885 10.3390/pharmaceutics13020152 PMC7911720

[CR50] Montalvo-Quirós S, Gómez-Graña S, Vallet-Regí M, Prados-Rosales RC, González B, Luque-Garcia JL (2021) Mesoporous silica nanoparticles containing silver as novel antimycobacterial agents against *Mycobacterium tuberculosis*. Colloids Surf B Biointerfaces 197:111405. 10.1016/j.colsurfb.2020.111405 33130523 10.1016/j.colsurfb.2020.111405

[CR51] Morgan RN, Aboshanab KM (2024) Green biologically synthesized metal nanoparticles: biological applications, optimizations and future prospects. Future Sci OA 10(1):FSO935. 10.2144/fsoa-2023-0196 38817383 10.2144/fsoa-2023-0196 PMC11137799

[CR52] Nie P, Zhao Y, Xu H (2023) Synthesis, applications, toxicity and toxicity mechanisms of silver nanoparticles: a review. Ecotoxicol Environ Saf 253:114636. 10.1016/j.ecoenv.2023.114636 36806822 10.1016/j.ecoenv.2023.114636

[CR53] Pasquet J, Chevalier Y, Pelletier J, Couval E, Bouvier D, Bolzinger M-A (2014) The contribution of zinc ions to the antimicrobial activity of zinc oxide. Colloids Surf Physicochem Eng Asp 457:263–274. 10.1016/j.colsurfa.2014.05.057

[CR54] Pulingam T, Parumasivam T, Gazzali AM, Sulaiman AM, Chee JY, Lakshmanan M, Chin CF, Sudesh K (2022) Antimicrobial resistance: prevalence, economic burden, mechanisms of resistance and strategies to overcome. Eur J Pharm Sci 170:106103. 10.1016/j.ejps.2021.106103 34936936 10.1016/j.ejps.2021.106103

[CR55] Raghunath A, Perumal E (2017) Metal oxide nanoparticles as antimicrobial agents: a promise for the future. Int J Antimicrob Agents 49:137–152. 10.1016/j.ijantimicag.2016.11.011 28089172 10.1016/j.ijantimicag.2016.11.011

[CR56] Saif MS, Zafar A, Waqas M, Hassan SG, Haq A, ul, Tariq T, Batool S, Dilshad M, Hasan M, Shu X (2021) Phyto-reflexive zinc oxide Nano-Flowers synthesis: an advanced photocatalytic degradation and infectious therapy. J Mater Res Technol 13:2375–2391. 10.1016/j.jmrt.2021.05.107

[CR57] Saif MS, Hasan M, Zafar A, Ahmed MM, Tariq T, Waqas M, Hussain R, Zafar A, Xue H, Shu X (2023) Advancing nanoscale science: synthesis and bioprinting of zeolitic imidazole framework-8 for enhanced anti-infectious therapeutic efficacies. Biomedicines. 10.3390/biomedicines11102832 37893205 10.3390/biomedicines11102832 PMC10604899

[CR58] Salleh A, Naomi R, Utami ND, Mohammad AW, Mahmoudi E, Mustafa N, Fauzi MB (2020) The potential of silver nanoparticles for antiviral and antibacterial applications: a mechanism of action. Nanomaterials 10:1566. 10.3390/nano10081566 32784939 10.3390/nano10081566 PMC7466543

[CR59] Salmani-Zarchi H, Mousavi-Sagharchi SMA, Sepahdoost N, Ranjbar-Jamalabadi M, Gross JD, Jooya H, Samadi A (2024) Antimicrobial feature of nanoparticles in the antibiotic resistance era: from mechanism to application. Adv Biomed Res 13. 10.4103/abr.abr_92_24 10.4103/abr.abr_92_24 PMC11665187 39717242

[CR60] Sarkar S, Roy A, Mitra R, Kundu S, Banerjee P, Acharya Chowdhury A, Ghosh S (2024) Escaping the ESKAPE pathogens: a review on antibiofilm potential of nanoparticles. Microb Pathog 194:106842. 10.1016/j.micpath.2024.106842 39117012 10.1016/j.micpath.2024.106842

[CR61] Shen Z, Zhou H, Chen H, Xu H, Feng C, Zhou X (2018) Synthesis of nano-zinc oxide loaded on mesoporous silica by coordination effect and its photocatalytic degradation property of methyl orange. Nanomaterials. 10.3390/nano8050317 29747457 10.3390/nano8050317 PMC5977331

[CR62] Singh D, Sharma D, Agarwal V (2021) Screening of anti-microbial, anti-biofilm activity, and cytotoxicity analysis of a designed polyherbal formulation against shigellosis. J Ayurveda Integr Med 12:601–606. 10.1016/j.jaim.2021.06.007 34772585 10.1016/j.jaim.2021.06.007 PMC8642668

[CR63] Staroń A, Długosz O (2021) Antimicrobial properties of nanoparticles in the context of advantages and potential risks of their use. J Environ Sci Health A 56:680–693. 10.1080/10934529.2021.1917936 10.1080/10934529.2021.1917936 33979267

[CR64] Tang KWK, Millar BC, Moore JE (2023) Antimicrobial resistance (AMR). Br J Biomed Sci. 10.3389/bjbs.2023.11387 37448857 10.3389/bjbs.2023.11387 PMC10336207

[CR65] Tavakoli F, Mamaghani M, Sheykhan M (2019) Introduction of Ag/CuO/MCM-48 as an efficient catalyst for the one‐pot synthesis of novel pyran‐pyrrole hybrids. Appl Organomet Chem. 10.1002/aoc.5083

[CR66] Tian D, Chen Y, Lu X, Ling Y, Lin B (2021) Facile preparation of mesoporous MCM-48 containing silver nanoparticles with fly ash as raw materials for CO catalytic oxidation. Micromachines. 10.3390/mi12070841 34357251 10.3390/mi12070841 PMC8305745

[CR67] Ugalde-Arbizu M, Aguilera-Correa JJ, San Sebastian E, Páez PL, Nogales E, Esteban J, Gómez-Ruiz S (2023) Antibacterial properties of mesoporous silica nanoparticles modified with fluoroquinolones and copper or silver species. Pharmaceuticals. 10.3390/ph16070961 37513873 10.3390/ph16070961 PMC10386262

[CR68] van Hengel IAJ, Putra NE, Tierolf MWAM, Minneboo M, Fluit AC, Fratila-Apachitei LE, Apachitei I, Zadpoor AA (2020) Biofunctionalization of selective laser melted porous titanium using silver and zinc nanoparticles to prevent infections by antibiotic-resistant bacteria. Acta Biomater 107:325–337. 10.1016/j.actbio.2020.02.044 32145392 10.1016/j.actbio.2020.02.044

[CR69] Venkatathri N (2007) Synthesis of silica nanosphere from homogeneous and heterogeneous systems. Bull Mater Sci 30:615–617. 10.1007/s12034-007-0097-3

[CR70] Wan Mohtar WHM, Mohd Razali MA, Mazlan MA, Ahmad Rozaini AZ, Mooralitharan SAP, Abdul Hamid A, Buyong MR (2023) Rapid detection of ESKAPE and enteric bacteria using tapered dielectrophoresis and their presence in urban water cycle. Process Saf Environ Prot 177:427–435. 10.1016/j.psep.2023.06.088

[CR71] WHO (World Health Organization) (2023) (Antimicrobial resistance. In: 2023. https://www.who.int/news-room/fact-sheets/detail/antimicrobial-resistance (accessed on 27 June 2025)

[CR72] Wu H, Tian H, Li J, Liu L, Wang Y, Qiu J, Wang S, Liu S (2020) Self-detoxifying hollow zinc silica nanospheres with tunable Ag ion release-recapture capability: a nanoantibiotic for efficient MRSA inhibition. Compos B Eng 202:108415. 10.1016/j.compositesb.2020.108415

[CR73] Yao JH, Ortega EF, Panda A (2025) Impact of zinc on immunometabolism and its putative role on respiratory diseases. Immunometabolism 7(1):e00057. 10.1097/in9.0000000000000057 40051614 10.1097/IN9.0000000000000057 PMC11882175

[CR74] Yismaw S, Wenzel M, Attallah AG, Zaleski R, Matysik J, Poppitz D, Gläser R, Ebbinghaus SG, Enke D (2023) Core-shell structured MCM-48-type silica-polymer hybrid material synthesis and characterization. J Nanopart Res. 10.1007/s11051-022-05666-2

[CR75] Zhang X, Hou Y, Huang Y, Chen W, Zhang H (2023) Interplay between zinc and cell proliferation and implications for the growth of livestock. J Anim Physiol Anim Nutr (Berl) 107:1402–1418. 10.1111/jpn.13851 37391879 10.1111/jpn.13851

[CR76] Zhu Y, Huang WE, Yang Q (2022) Clinical perspective of antimicrobial resistance in bacteria. Infect Drug Resist 15:735–746. 10.2147/IDR.S345574 35264857 10.2147/IDR.S345574 PMC8899096

