# Supplementary material for: Antibacterial, antibiofilm and cytotoxic activity of synthesized metal-incorporated mesoporous silica nanoparticles
Source: AMB Express. 2025 Sep 17;15:130. doi: 10.1186/s13568-025-01938-x (PMC12443651; doi:10.1186/s13568-025-01938-x)
Supplement: Supplementary file 1 — Supplementary Material 1 [file 13568_2025_1938_MOESM1_ESM.docx]

**Fig. S1**. Schematic diagram of the whole methodology

**Fig. S2.** The recorded inhibition zone **(**antibacterial activity) of (1) MCM-48 NPs and (3) Ag/MCM-48 NPs (10 mmol) against *Escherichia coli* 48 WE, (C): Gentamicin 4 µg/ mL

**Fig. S3.** The recorded inhibition zone **(**antibacterial activity) of (2) Ag/Zn/MCM-48 NPs 10 mmol and (3) Ag/MCM-48 NPs (10 mmol) against *Escherichia coli* 48 WE, (C): Ciprofloxacin 10 µg/ mL

**Fig. S4.** The recorded inhibition zone **(**antibacterial activity) of (1) MCM-48 NPs and (3) Ag/MCM-48 NPs (10 mmol) against *Klebsiella pneumoniae* 36 AK, (C): Ciprofloxacin 10 µg/ mL.

**Fig. S5**. The recorded inhibition zone **(**antibacterial activity) of (2) Ag/Zn/MCM-48 NPs (10 mmol) and (3) Ag/MCM-48 NPs against *Klebsiella pneumoniae* 36 AK*,* (C): Ciprofloxacin 10 µg/ ml

**Fig. S6**. The recorded inhibition zone **(**antibacterial activity) of (1) Ag/Zn/MCM-48 NPs (10 mmol) and (2) Ag/MCM-48 NPs (10 mmol) against MRSA ATCC 33591, (B): sterilized distilled water, (C): Ciprofloxacin 10 µg/ mL.

**Fig. S7**. The recorded inhibition zone **(**antibacterial activity) of (1) Ag/Zn/MCM-48 NPs (10 mmol) and (2) Ag/MCM-48 NPs (10 mmol) against *Escherichia coli* 25 LN*,* (B): sterilized distilled water, (C): Ciprofloxacin 10 µg/ mL.

**Fig. S8.** The recorded inhibition zone **(**antibacterial activity) of (1) Ag/Zn/MCM-48 NPs (10 mmol) and (2) Ag/MCM-48/ NPs (10 mmol) against *Klebsiella pneumoniae* 78 TE, (B): sterilized distilled water, (C): Ciprofloxacin 10 µg/ mL.

**Fig. S9**. The recorded inhibition zone **(**antibacterial activity) of (1) Ag/Zn/MCM-48 NPs (10 mmol) and (2) Ag/MCM-48 NPs (10 mmol) against *Salmonella* Typhi 24 ZA*,* (B): sterilized distilled water, (C): Ciprofloxacin 10 µg/ mL.

**Table S1.** The viability of *Vero* cell line with Ag/MCM-48 10 mmol nanoparticles**.**

| **Ag/MCM-48 NPs' conc. (µg/ml)** | **Viability %** | **Inhibitory %** | **Standard deviation** |
| --- | --- | --- | --- |
| 500 | 2.34 | 97.66 | 0.12 |
| 250 | 6.97 | 93.03 | 0.31 |
| 125 | 13.68 | 86.32 | 0.44 |
| 62.5 | 23.89 | 76.11 | 0.97 |
| 31.25 | 42.31 | 57.69 | 1.73 |
| 15.6 | 52.97 | 47.03 | 1.05 |
| 7.8 | 64.18 | 35.82 | 2.34 |
| 3.9 | 81.29 | 18.71 | 1.65 |
| 2 | 88.67 | 11.33 | 0.79 |
| 1 | 94.21 | 5.79 | 0.33 |
| 0 | 100 | 0 |  |

**Table S2.** The viability of the *Vero* ell line with Ag/Zn/MCM-48 (10 mmol) nanoparticles**.**

| **Ag/Zn/MCM-48 NPs' conc. (µg/ml)** | **Viability %** | **Inhibitory %** | **Standard deviation** |
| --- | --- | --- | --- |
| 500 | 17.53 | 82.47 | 1.95 |
| 250 | 38.96 | 61.04 | 1.28 |
| 125 | 56.03 | 43.97 | 1.41 |
| 62.5 | 76.41 | 23.59 | 1.23 |
| 31.25 | 93.69 | 6.31 | 0.07 |
| 15.6 | 99.52 | 0.48 | 0.14 |
| 7.8 | 100 | 0 |  |
| 3.9 | 100 | 0 |  |
| 2 | 100 | 0 |  |
| 1 | 100 | 0 |  |
| 0 | 100 | 0 |  |

**Table S3**. The viability of the *Caco*-2 cell line with Ag/MCM-48 10 mmol nanoparticles.

| **Ag/MCM-48 NPs' conc. (µg/ml)** | **Viability %** | **Inhibitory %** | **S.D. (standard deviation)** |
| --- | --- | --- | --- |
| 500 | 0.79 | 99.21 | 0.23 |
| 250 | 3.18 | 96.82 | 0.16 |
| 125 | 6.72 | 93.28 | 0.24 |
| 62.5 | 11.48 | 88.52 | 0.64 |
| 31.25 | 16.23 | 83.77 | 0.31 |
| 15.6 | 21.69 | 78.31 | 0.75 |
| 7.8 | 29.74 | 70.26 | 1.08 |
| 3.9 | 43.81 | 56.19 | 2.73 |
| 2 | 72.95 | 27.05 | 1.91 |
| 1 | 80.63 | 19.37 | 0.71 |
| 0 | 100 | 0 |  |

**Table S4**. The viability of the *Caco*-2 cell line with Ag/Zn/MCM-48 10 mmol nanoparticles.

| **Ag/Zn/MCM-48 NPs' conc. (µg/ml)** | **Viability %** | **Inhibitory %** | **S.D. (standard deviation)** |
| --- | --- | --- | --- |
| 500 | 8.49 | 91.51 | 1.23 |
| 250 | 19.51 | 80.49 | 1.75 |
| 125 | 32.85 | 67.15 | 2.31 |
| 62.5 | 49.70 | 50.3 | 1.84 |
| 31.25 | 78.23 | 21.77 | 1.95 |
| 15.6 | 92.74 | 7.26 | 0.62 |
| 7.8 | 98.57 | 1.43 | 0.37 |
| 3.9 | 100 | 0 |  |
| 2 | 100 | 0 |  |
| 1 | 100 | 0 |  |
| 0 | 100 | 0 |  |
